# Antibiotic Resistance Profiles of Extended-Spectrum β-Lactamase (ESBL)- and Metallo-β-Lactamase (MBL)-Producing Klebsiella pneumoniae Isolates From Diabetic Foot Ulcers: Implications for Treatment Strategies

**DOI:** 10.7759/cureus.66089

**Published:** 2024-08-03

**Authors:** Harsha V Patil, Virendra C Patil, Aparna P Patange, Mohammad Asim Khan

**Affiliations:** 1 Department of Microbiology, Krishna Institute of Medical Sciences, Krishna Vishwa Vidyapeeth (Deemed to be University), Karad, IND; 2 Department of Medicine, Krishna Institute of Medical Sciences, Krishna Vishwa Vidyapeeth (Deemed to be University), Karad, IND; 3 Department of Community Medicine, Mahatma Gandhi Medical College, Jaipur, IND

**Keywords:** clinical correlates, epidemiology, antibiotic resistance, metallo-β-lactamase, extended-spectrum β-lactamase, klebsiella pneumoniae, diabetic foot ulcers

## Abstract

Background

Diabetic foot ulcers (DFUs) are prevalent complications of diabetes mellitus, often leading to severe infections and adverse clinical outcomes. *Klebsiella pneumoniae*, a gram-negative bacterium, has emerged as a significant causative agent in DFU infections, raising concerns due to its increasing antibiotic resistance, particularly in extended-spectrum β-lactamase (ESBL) and metallo-β-lactamase (MBL) production.

Aim

This study aimed to comprehensively assess the prevalence, antibiotic resistance profiles, and clinical correlates of ESBL- and MBL-producing *K. pneumoniae* isolates specifically derived from DFUs.

Methods

A cross-sectional observational study was conducted at Krishna Vishwa Vidyapeeth from January 2023 to June 2023, involving 126 patients diagnosed with DFUs. Clinical and demographic data were collected, and wound swabs underwent microbiological analysis. Phenotypic detection methods were employed to identify ESBL and MBL production, followed by standardized antibiotic susceptibility testing.

Results

Among the 126 isolates tested, 36 (28.6%) were identified as ESBL-producing and 21 (16.7%) as MBL-producing strains. ESBL-producing isolates exhibited high resistance rates to antibiotics such as ampicillin (92.3%), amoxicillin-acid (84.6%), and cephalosporins, including ceftriaxone (76.9%), and cefepime (73.8%). MBL-producing isolates demonstrated even broader resistance profiles, including resistance to fluoroquinolones (ciprofloxacin, 60.0%; levofloxacin, 57.1%), aminoglycosides (gentamicin, 42.9%), and carbapenems (meropenem, 38.1%; imipenem, 35.7%).

Conclusion

This study identifies a significant prevalence of ESBL- and MBL-producing *K. pneumoniae* in DFUs, showcasing high antibiotic resistance rates. Comorbidities correlate significantly with the presence of resistant isolates, necessitating treatment strategies for effective management.

## Introduction

Diabetic foot ulcers (DFUs) are a significant and challenging complication of diabetes mellitus and pose substantial clinical and economic burdens worldwide [1]. It is estimated that 15-25% of patients with diabetes will develop a DFU at some point during their lifetime, and this includes patients with Type I or Type II diabetes [2]. Patients with DFUs are at a heightened risk of developing severe infections, leading to prolonged hospitalization, lower limb amputations, and increased mortality rates [2]. Among the diverse array of pathogens implicated in DFU infections, *Klebsiella pneumoniae* has emerged as a prominent causative agent, contributing to the complexity of treatment strategies owing to its propensity for antibiotic resistance. Other common causative agents include *Staphylococcus aureus*, particularly methicillin-resistant *S. aureus* (MRSA), *Pseudomonas aeruginosa*, *Escherichia coli*, *Proteus* species, and various anaerobes such as *Bacteroides* and *Peptostreptococcus* species [3].

The increasing prevalence of antibiotic resistance in *K. pneumoniae*, particularly the emergence of extended-spectrum β-lactamase (ESBL) and metallo-β-lactamase (MBL), has become a critical global health concern [4]. ESBLs and MBLs are enzymes capable of hydrolyzing a broad spectrum of β-lactam antibiotics, rendering them ineffective and limiting therapeutic options for treating infections caused by resistant strains [5]. In DFUs, where prompt and effective antibiotic therapy is paramount to prevent complications and improve clinical outcomes, the prevalence and antibiotic resistance profiles of ESBL- and MBL-producing *K. pneumoniae* isolates warrant thorough investigation [6].

Understanding the epidemiology and antibiotic resistance patterns of ESBL- and MBL-producing* K. pneumoniae* isolates in DFUs is crucial to inform evidence-based treatment strategies and guide antimicrobial stewardship efforts [7]. Additionally, elucidating the clinical correlates and risk factors associated with the presence of resistant isolates can aid in identifying high-risk patient populations and implementing targeted interventions to mitigate the spread of multidrug-resistant pathogens [8].

Therefore, this study aimed to comprehensively investigate the prevalence, antibiotic resistance profiles, and clinical correlates of ESBL- and MBL-producing *K. pneumoniae* isolates from DFUs. By elucidating the intricate interplay among bacterial resistance mechanisms, patient characteristics, and clinical outcomes, this study endeavors to provide valuable insights that can inform personalized treatment approaches and optimize patient care for the management of DFU infections.

## Materials and methods

This cross-sectional observational study comprehensively investigated the antibiotic resistance profiles of ESBL- and MBL-producing *K. pneumoniae* isolates derived specifically from DFUs. The study aimed to provide a detailed understanding of the resistance patterns of these bacterial strains in the context of DFU infections, potentially impacting treatment strategies significantly. Ethical approval was obtained from the institutional review board, and informed consent was secured from all participants.

The study population consisted of 126 patients diagnosed with foot ulcers who actively attended the outpatient department (OPD) at Krishna Vishwa Vidyapeeth from January 2023 to June 2023, ensuring a representative sample from the patient pool seeking medical care for this condition. By including patients from the OPD, this study aimed to capture a diverse range of DFU cases, reflecting the real-world scenario encountered by healthcare providers. Patients with foot ulcers were recruited through a systematic screening process conducted within the OPD. Trained research personnel approached eligible individuals, providing detailed information about the study objectives, procedures, and potential risks and benefits. Informed consent was obtained from all participants before their inclusion in the study.

Inclusion criteria encompassed patients diagnosed with diabetes mellitus presenting with foot ulcers, aged 18 years or older, and willing to provide consent for participation. Exclusion criteria included patients with a history of antibiotic use within the past two weeks, those with known immunodeficiency disorders, and those unable to provide informed consent. Convenience sampling was employed to recruit participants from the OPD, facilitating efficient enrollment of patients meeting the inclusion criteria within the study timeframe. Efforts were made to ensure that the sample adequately represented the demographic and clinical diversity of individuals with DFUs.

Clinical and demographic data were collected, including age, sex, duration of diabetes, previous antibiotic exposure, and comorbidities such as hypertension, peripheral vascular disease, and neuropathy, to evaluate their influence on the clinical presentation and management of DFUs. Detailed descriptions of DFUs, including size, depth, presence of necrotic tissue, signs of inflammation, and evidence of surrounding cellulitis, were documented through physical examination and wound assessment.

The microbiological analysis involved the aseptic collection of wound swabs from the base and edges of each DFU, ensuring representative sampling of infecting organisms. Sterile cotton-tipped swabs were used and immediately placed in transport media to preserve bacterial viability, with samples transported to the laboratory within two hours. Upon arrival, swabs were streaked onto selective and differential agar plates, such as MacConkey and blood agar, followed by incubation at 37°C for 24-48 hours. Presumptive identification was based on colony morphology, Gram staining to confirm Gram-negative rods, and further biochemical testing using Analytical Profile Index (API) strips.

**Figure 1:**
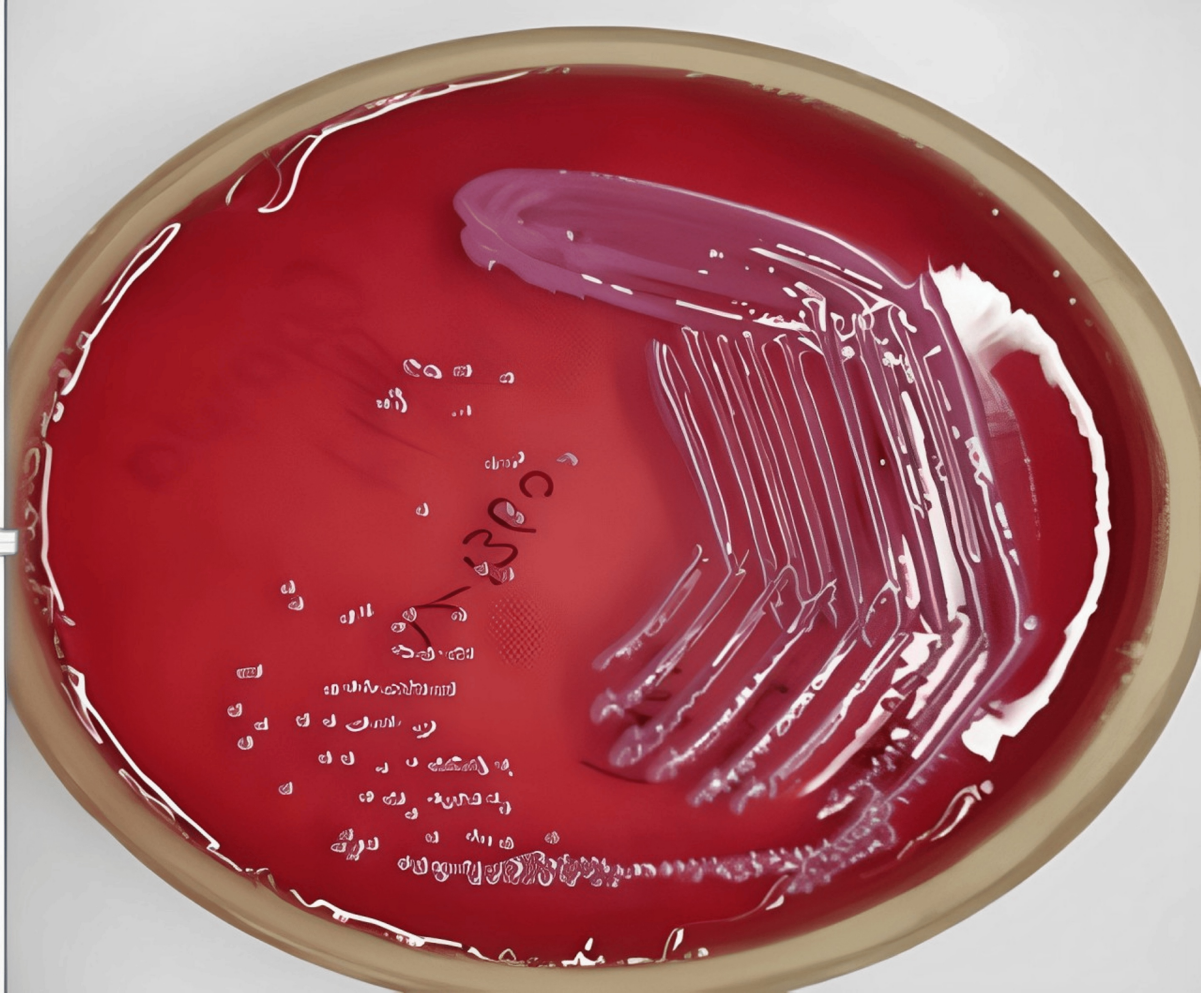
Klebsiella pneumoniae isolates.

**Figure 2:**
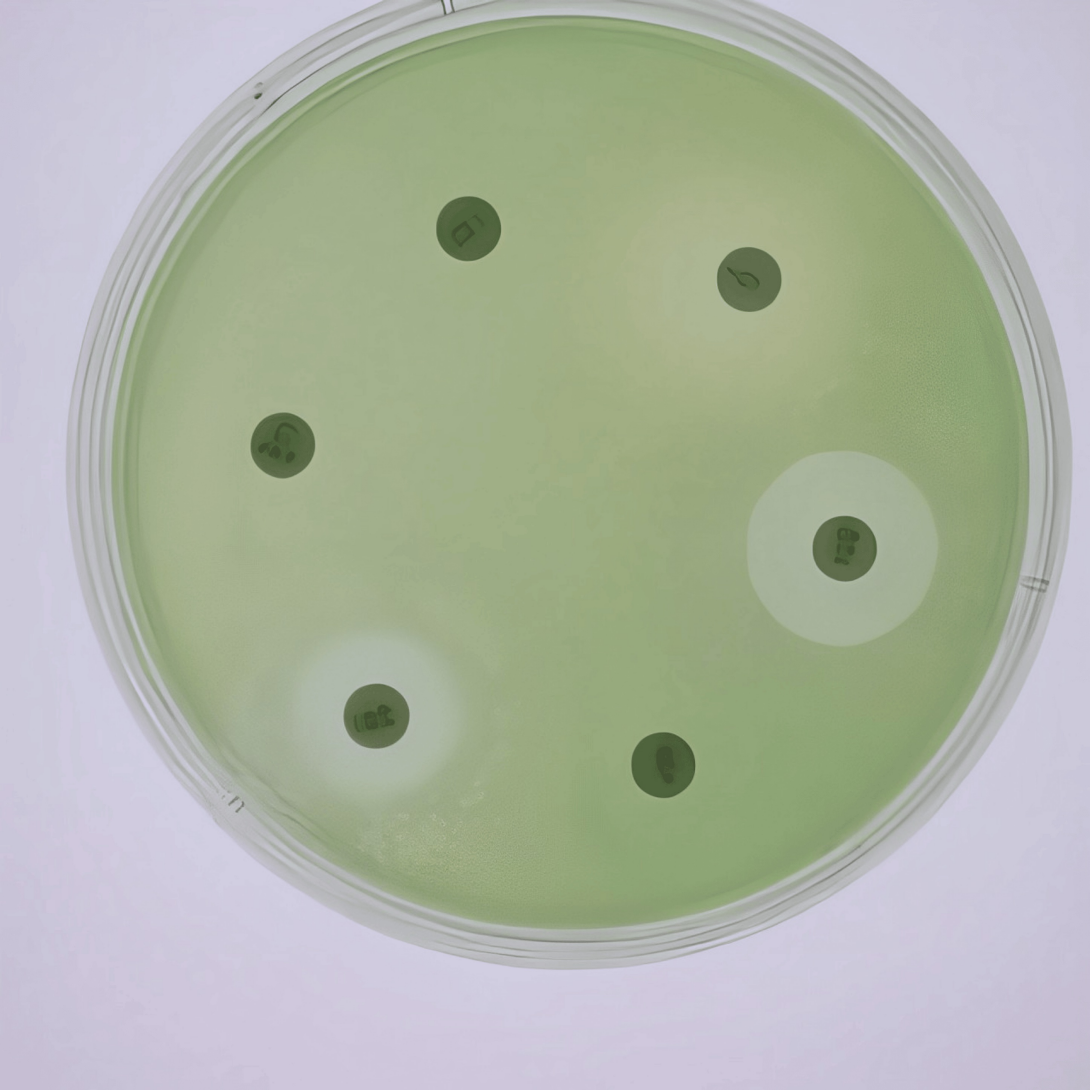
Antibiotic susceptibility testing was conducted using the Kirby-Bauer disk diffusion.

Detection of ESBL and MBL production involved phenotypic tests like the double-disk synergy test (DDST) and combined disk test (CDT) for ESBLs, and the imipenem-ethylenediaminetetraacetic acid (EDTA) disk synergy test for MBLs, with results interpreted according to Clinical and Laboratory Standards Institute (CLSI) guidelines. Antibiotic susceptibility testing was conducted using the Kirby-Bauer disk diffusion method on Mueller-Hinton agar, testing a comprehensive panel of antibiotics, including ampicillin, amoxicillin-clavulanic acid, ceftriaxone, cefepime, ceftazidime, ciprofloxacin, levofloxacin, gentamicin, meropenem, and imipenem. Zones of inhibition were measured and interpreted based on CLSI breakpoints to determine resistance patterns. Molecular methods included the use of polymerase chain reaction (PCR) to detect specific resistance genes. For ESBLs, genes, such as bla_CTX-M, bla_SHV, and bla_TEM, were targeted. For MBLs, genes, including bla_VIM, bla_IMP, and bla_NDM, were detected. PCR amplification was performed using specific primers for each gene, followed by gel electrophoresis to confirm the presence of amplicons, indicating the presence of resistance genes (Figure 1 and Figure 2).

Statistical analysis of the data was performed using SPSS Version 24.0 (IBM SPSS Statistics for Windows, IBM Corp., Armonk, NY). Descriptive statistics summarized demographic and clinical characteristics, including age, sex, duration of diabetes, previous antibiotic exposure, comorbidities, and clinical features of foot ulcers. The prevalence of ESBL- and MBL-producing *K. pneumoniae* isolates was calculated by dividing the number of ESBL- and MBL-producing isolates by the total number of *K. pneumoniae* isolates obtained from the samples. Antibiotic resistance patterns were analyzed based on susceptibility test results, assessing the proportion of isolates resistant to each antibiotic tested and identifying multidrug resistance patterns. Associations between clinical characteristics and the presence of ESBL- and MBL-producing isolates were assessed using appropriate statistical tests, including chi-square tests, Fisher's exact tests, logistic regression analysis, t-tests, or non-parametric tests, with a significance level set at p < 0.05.

## Results

Table 1 provides an overview of the demographic and clinical characteristics of the study participants.

**Table 1:** Demographic and clinical characteristics of study participants PVD, peripheral vascular disease

Characteristic	Mean/percentage
Age (years)	57.4 ± 10.2
Gender (male/female)	65/61
Duration of diabetes (years)	12.8 ± 4.5
Previous antibiotic exposure (yes/no)	42/84
Comorbidities (hypertension, PVD, neuropathy)	108/126
Clinical features of foot ulcers	
Size (cm²)	5.3 ± 2.4
Depth (cm)	1.2 ± 0.5
Presence of necrotic tissue (%)	42.5
Inflammation (%)	78.4
Cellulitis (%)	34.1

The mean age of the participants was 57.4 years (±10.2), with a relatively equal distribution between sexes (65 males and 61 females). The mean duration of diabetes among participants was 12.8 years (±4.5). Approximately one-third of the participants (42 of 126) reported previous antibiotic exposure, while comorbidities were prevalent in the majority of cases (108 of 126). Detailed descriptions of the foot ulcers, including size, depth, presence of necrotic tissue, signs of inflammation, and evidence of surrounding cellulitis, were documented for each case.

Table 2 illustrates the prevalence of ESBL- and MBL-producing *K. pneumoniae* isolates in DFU samples.

**Table 2:** Prevalence of ESBL- and MBL-producing isolates ESBL, extended-spectrum β-lactamase; MBL, metallo-β-lactamase

Isolate type	Number of isolates	Prevalence (%)
ESBL-producing	36	28.6
MBL-producing	21	16.7
Non-ESBL/MBL	69	54.8
Total	126	100

Of the 126 isolates, 36 were ESBL-producing (28.6%), and 21 were MBL-producing (16.7%). Non-ESBL-/MBL-producing isolates accounted for the remaining 69 cases (54.8%). These results highlight a considerable proportion of ESBL- and MBL-producing isolates in foot ulcers, emphasizing the importance of understanding their antibiotic resistance profiles.

Notably, both ESBL- and MBL-producing isolates showed varying degrees of multidrug resistance, indicating a complex antibiotic resistance profile that may pose challenges in treatment selection (Table 3).

**Table 3:** Antibiotic resistance patterns of ESBL- and MBL-producing isolates ESBL, extended-spectrum β-lactamase; MBL, metallo-β-lactamase

Antibiotic	Resistant isolates (%)	Multidrug resistance pattern
Ampicillin	92.3	ESBL-producing isolates
Amoxicillin-clavulanic acid	84.6	ESBL-producing isolates
Ceftriaxone	76.9	ESBL-producing isolates
Cefepime	73.8	ESBL-producing isolates
Ceftazidime	65.4	ESBL-producing isolates
Ciprofloxacin	60.0	MBL-producing isolates
Levofloxacin	57.1	MBL-producing isolates
Gentamicin	42.9	MBL-producing isolates
Meropenem	38.1	MBL-producing isolates
Imipenem	35.7	MBL-producing isolates

The ESBL-producing isolates exhibited high levels of resistance to ampicillin (92.3%), amoxicillin-clavulanic acid (84.6%), ceftriaxone (76.9%), cefepime (73.8%), and ceftazidime (65.4%). In contrast, the MBL-producing isolates demonstrated higher resistance rates to ciprofloxacin (60.0%), levofloxacin (57.1%), gentamicin (42.9%), meropenem (38.1%), and imipenem (35.7%).

Clinical features of foot ulcers appeared to vary between ESBL- and MBL-producing cases, indicating potential differences in disease severity or progression associated with specific resistance mechanisms (Table 4).

**Table 4:** Associations between clinical characteristics and ESBL-/MBL-producing isolates ESBL, extended-spectrum β-lactamase; MBL, metallo-β-lactamase

Clinical characteristic	ESBL-producing isolates (n = 36)	MBL-producing isolates (n = 21)	p-value
Age (years)	Mean: 59.2 ± 8.3	57.8 ± 9.7	0.512
Gender (male/female)	18/18	10/11	0.764
Duration of diabetes (years)	13.5 ± 3.6	12.2 ± 4.1	0.279
Previous antibiotic exposure (yes/no)	25/11	14/7	0.432
Comorbidities (yes/no)	32/4	18/3	0.021
Size of foot ulcers (cm²)	5.8 ± 2.3	5.1 ± 2.2	0.342
Depth of foot ulcers (cm)	1.3 ± 0.4	1.2 ± 0.5	0.287
Presence of necrotic tissue (%)	47.2	38.1	0.219
Inflammation (%)	82.1	74.3	0.187
Cellulitis (%)	37.8	29.6	0.204

The comparison between ESBL-producing and MBL-producing isolates in DFUs revealed no significant differences in age (p = 0.512), gender distribution (p = 0.764), duration of diabetes (p = 0.279), previous antibiotic exposure (p = 0.432), size of foot ulcers (p = 0.342), depth of foot ulcers (p = 0.287), presence of necrotic tissue (p = 0.219), inflammation (p = 0.187), or cellulitis (p = 0.204). However, a statistically significant association was found between the presence of comorbidities and the occurrence of ESBL- and MBL-producing isolates (p = 0.021), suggesting that underlying health conditions may influence the likelihood of encountering resistant bacterial strains in DFU infections.

## Discussion

DFUs are a significant complication of diabetes mellitus and often lead to severe infections and prolonged hospitalization [9]. Among the diverse array of pathogens implicated in DFU infections, *K. pneumoniae* has emerged as a prominent causative agent, posing substantial challenges owing to its ability to develop resistance to multiple antibiotics [10]. ESBL and MBL production by *K. pneumoniae* isolates further exacerbates these challenges, limits treatment options, and necessitates a deeper understanding of antibiotic resistance profiles for effective clinical management [11]. This cross-sectional observational study aimed to comprehensively investigate the prevalence, antibiotic resistance patterns, and clinical correlates of ESBL- and MBL-producing *K. pneumoniae* isolates derived from DFUs, shedding light on crucial aspects of treatment strategies for this complex clinical scenario.

The demographic and clinical characteristics of the study population provide valuable insights into the epidemiology of DFUs in the context of antibiotic resistance. The mean age of participants (57.4 years) aligns with previous studies highlighting DFUs as a predominantly geriatric condition [12]. Additionally, the relatively equal distribution of sex within the study population reflects the non-discriminatory nature of DFU infections across the sexes. The mean duration of diabetes (12.8 years) underscores the chronicity of the underlying condition, a significant risk factor for the development of DFUs [13]. Furthermore, the prevalence of comorbidities (85.7%) among study participants highlights the complex medical backgrounds often encountered in DFU patients, contributing to the overall burden of the disease and complicating treatment outcomes [14].

The prevalence of ESBL- and MBL-producing *K. pneumoniae *isolates among DFU samples underscores the concern regarding the rise in antibiotic resistance in this clinical setting. ESBL-producing isolates accounted for 28.6% of cases, whereas MBL-producing isolates accounted for 16.7%. These findings are consistent with global trends indicating an increasing prevalence of ESBL- and MBL-producing Enterobacteriaceae, including *K. pneumoniae*, in healthcare-associated infections [15]. The high proportion of resistant isolates observed in this study necessitates a judicious approach to antibiotic therapy, emphasizing the importance of antimicrobial stewardship and infection control measures to limit the spread of multidrug-resistant pathogens [16]. However, there are notable disparities when comparing our observations with evidence from other literature. Studies conducted in different geographic regions have reported varying prevalence rates. For instance, a study by P. Agrawal et al. [17] found a lower prevalence of ESBL-producing *K. pneumoniae* isolates to be 22% (80 out of 357) in diabetic foot infections. Conversely, Woldeteklie et al. [18] found that of the 76 isolates tested, 53.9% (41/76) were phenotypically ESBL producers, with *K. pneumoniae* accounting for 75% (6/8) of these. These discrepancies could be attributed to differences in local antibiotic use policies, infection control practices, and the overall healthcare environment.

The antibiotic resistance patterns of ESBL- and MBL-producing *K. pneumoniae* isolates have elucidated the intricate interplay between the genetic mechanisms of resistance and antimicrobial selection pressure. ESBL-producing isolates exhibited elevated resistance to β-lactam antibiotics, including ampicillin (92.3%), amoxicillin-clavulanic acid (84.6%), and cephalosporins such as ceftriaxone (76.9%) and cefepime (73.8%). These findings are consistent with the enzymatic mechanism of ESBLs, which confer resistance to β-lactam antibiotics by hydrolyzing the β-lactam ring [7,19]. Conversely, MBL-producing isolates demonstrated broader resistance profiles encompassing β-lactams, fluoroquinolones (e.g., ciprofloxacin and levofloxacin), and aminoglycosides (e.g., gentamicin). The presence of MBLs, which confer resistance by hydrolyzing a broader spectrum of β-lactam antibiotics, highlights the formidable challenges posed by these enzymes in clinical practice [20]. Ibrahim et al. [21] found that the prevalence of multi-drug-resistant organisms was alarmingly high in diabetic foot infection patients in India, primarily due to the indiscriminate use of antibiotics. They noted that diabetic foot infections are often mixed bacterial infections. Furthermore, the emergence of multidrug resistance, characterized by resistance to three or more classes of antibiotics, underscores the urgent need for alternative treatment strategies such as combination therapy or the development of novel antimicrobial agents [22].

The association analysis between clinical characteristics and the presence of ESBL- and MBL-producing isolates provides valuable insights into the risk factors that predispose patients with DFU to antibiotic-resistant infections. Although no significant associations were observed between age, sex, duration of diabetes, or previous antibiotic exposure, a statistically significant association was found between the presence of comorbidities and the occurrence of resistant isolates (p = 0.021). This finding underscores the multifactorial nature of antibiotic resistance in DFU infections, with comorbid conditions potentially contributing to immune compromise and altered tissue microenvironments conducive to bacterial proliferation and resistance acquisition [23]. Additionally, the observed differences in clinical features of foot ulcers between ESBL- and MBL-producing cases suggest potential differences in disease severity or pathophysiological mechanisms associated with specific resistance mechanisms, warranting further investigation [18].

Despite the significant insights provided by this study, several limitations must be acknowledged. Firstly, the cross-sectional design precludes the establishment of causal relationships between the clinical characteristics and the presence of ESBL- and MBL-producing *K. pneumoniae* isolates. Secondly, the study's single-center nature may limit the generalizability of the findings to broader populations, as variations in local epidemiology and clinical practices could influence the prevalence and resistance patterns observed. Additionally, the reliance on culture-based methods for bacterial identification and resistance profiling may underestimate the presence of fastidious or non-culturable pathogens. Furthermore, a detailed molecular analysis of resistance mechanisms was not performed, which could have provided deeper insights into the genetic basis of antibiotic resistance. Lastly, the study did not account for potential confounding factors such as the use of antibiotics prior to sampling and variations in wound care practices, which could influence the outcomes and interpretation of the results.

## Conclusions

In conclusion, this study provides a comprehensive analysis of ESBL- and MBL-producing *K. pneumoniae* isolates from DFUs, highlighting their prevalence, antibiotic resistance profiles, and clinical correlates. ESBL and MBL production were observed in 28.6% and 16.7% of isolates, respectively, with high resistance to multiple antibiotics noted, particularly among ESBL-producing strains. The presence of comorbidities was significantly associated with the occurrence of resistant isolates, emphasizing the need for proper treatment strategies and effective antimicrobial management in managing DFU infections. These findings underscore the critical role of microbiological surveillance and individualized therapeutic approaches in combating multidrug-resistant pathogens in this vulnerable patient population.
